# 2'‐Fucosyllactose attenuates aging‐related metabolic disorders through modulating gut microbiome‐T cell axis

**DOI:** 10.1111/acel.14343

**Published:** 2024-09-20

**Authors:** Ang Li, Ruixin Kou, Ruishan Wang, Jin Wang, Bowei Zhang, Jingmin Liu, Yaozhong Hu, Shuo Wang

**Affiliations:** ^1^ Tianjin Key Laboratory of Food Science and Health, School of Medicine Nankai University Tianjin China

**Keywords:** 2'‐Fucosyllactose, aging, gut microbiota, metabolic dysfunction, T cell

## Abstract

Aging‐related metabolic disorders seriously affect the lifespan of middle‐aged and older people, potentially due to disruptions in the adaptive immune and gut microbial profiles. Dietary intervention offers a promising strategy for maintaining metabolic health. This study aimed to investigate the ameliorative effect of 2′‐fucosyllactose (2'‐FL) on aging‐induced metabolic dysfunction and the underlying mechanisms. The results revealed that 2'‐FL significantly relieved aging‐related metabolic disorders, including weight gain, lipid deposition, dyslipidemia, glucose intolerance, systemic inflammation, and abnormal hepatic metabolism. Flow cytometry analysis revealed a significant reduction in T cytotoxic (Tc), T helper (Th), and regulatory T (Treg) cells and a significant increase in Th17 cells in aged mice, while 2'‐FL relieved the aging‐induced proportional changes in Th and Th17 subtypes. The aging intestinal microecology was characterized by higher Th17/Treg ratios, impaired gut barrier function, lower gut bacterial diversity, decreased abundance of beneficial genera including *Ligilactobacillus*, *Colidextribacter*, *Mucispirillum*, and *Lachnoclostridium*, and increased abundance of harmful bacteria including *Turicibacter* and *Desulfovibrio*, which was ameliorated by 2'‐FL treatment. These findings highlight that 2'‐FL is an ideal dietary prebiotic for improving aging‐related metabolic disorders by modulating both the adaptive immune system and the gut microbial profile.

## Graphical abstract

2'‐Fucosyllactose relieved aging‐related metabolic disorders by modulating both the adaptive immune system and gut microbial profile.

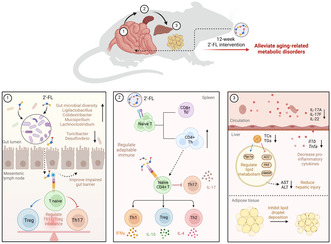

**Abbreviations**
2'‐FL: 2'‐Fucosyllactose
Acc: 1‐aminocyclopropane‐1‐carboxylic acid
ALT: alanine aminotransferase
AST: aspartate aminotransferase
AUC: area under the curve
FITC: fluorescein iso‐thiocyanate
H&E: hematoxylin and eosin
HDL‐C: high‐density lipoprotein cholesterol
Hmgr: HMG‐CoA reductase
HMOs: human milk oligosaccharides
IL‐17A: interleukin‐17A
IL‐17F: interleukin‐17F
IL‐22: interleukin‐22
KEGG: Kyoto Encyclopedia of Genes and Genomes
LDL‐C: low‐density lipoprotein cholesterol
LEfSe: linear discriminant analysis effect size
LPS: lipopolysaccharide
MLN: mesenteric lymph node
OGTT: oral glucose tolerance test
OTUs: operational taxonomic units
PCoA: principal co‐ordinates analysis
Pgc1a: PPAR‐gamma coactivator 1a
RT‐qPCR: real‐time quantitative polymerase chain reaction
Srebp1c: Sterol regulatory element‐binding protein 1C
Tc: T cytotoxic
TC: total cholesterol
TG: triglyceride
Th: T helper
Treg: T regulatory

## 1 INTRODUCTION

In the context of an aging population, the prevalence of obesity is increasing rapidly, with almost 35% of adults over the age of 65 considered obese (Fakhouri et al., 2012). Obesity in adulthood shortens life expectancy by about 7 years and is associated with multiple health issues (Tam et al., 2020). Notably, aging‐related weight gain and fat deposition due to confined metabolic efficiency, insufficient physical activity, and the accumulation of senescent cells could potentially lead to aging‐induced metabolic syndrome, including dyslipidemia, obesity, and type 2 diabetes (Hotamisligil, 2017). Accordingly, potential countermeasures to improve aging‐induced metabolic derangements are of great interest for healthy aging.

Aging‐related metabolic disorders are often characterized by persistent inflammation, which is strongly linked to immune dysfunction. Thus, an increasing number of studies have proposed a causal interpretation of immune senescence in aging‐associated disturbed metabolism (Carrasco et al., 2022). T lymphocytes, as critical regulators of adaptive immune responses, are more vulnerable to the aging process than innate immune components because thymic involution leads to a reduction in the number of both naïve and effector T cells exported to peripheral immune organs, and a decrease in the diversity of T‐cell receptors during aging (Mittelbrunn & Kroemer, 2021). This altered T‐cell profile may further disrupt the balance of pro‐ and anti‐inflammatory signals and contribute to aging‐induced metabolic dysregulation (Nikolich‐Zugich, 2018). Conventional T cells are categorized as CD8^+^ T cytotoxic (Tc) cells and CD4^+^ T helper (Th) cells, which are further classified as Th1, Th2, Th17, and T regulatory (Treg) subsets, depending on their functional properties and cytokine profiles. Of these, T cells tend to differentiate into pro‐inflammatory subtypes, including Th1 and Th17, and secrete pro‐inflammatory cytokines in aging, which are involved in the pathogenesis of aging‐driven metabolic diseases (Desdín‐Micó et al., 2020). Th17 differentiation has also been reported to be highly correlated with metabolic risk factors in a recent clinical trial (Phoksawat et al., 2020). Moreover, emerging evidence has shown that commensal gut microbes control whether naïve CD4^+^ T cells differentiate into either protective Treg cells or pro‐inflammatory Th17 cells (Petersen et al., 2019). Aging‐induced gut microflora dysbiosis, along with defects in the gastrointestinal barrier and loss of immune response, has been reported to exacerbate inflammatory injury and trigger metabolic disorders (Thevaranjan et al., 2017). Therefore, protective interventions targeting the aged gut microbiota structure may offer candidate therapies against aging‐related metabolic imbalances by shifting T cell differentiation toward non‐inflamed phenotypes. Diet is a well‐defined determinant of the gut microbiota composition, and oligosaccharides, one of the most frequently used prebiotics, have great potential to reshape the gut bacterial community (Chen & de Vos, 2023).

2‐Fucosyllactose (2'‐FL), a novel nondigestible carbohydrate derived from human milk oligosaccharides (HMOs), is composed of lactose (Galβ1‐4Glc) and fucose attached to the Gal terminal through an α1–2 bond. The prebiotic benefits of 2'‐FL have gained scientific attention recently, since 2'‐FL is known to exert functional properties by inhibiting colonic inflammation, modulating the production of short‐chain fatty acids, and regulating host immune responses with no reported adverse effects, and has been approved as a natural food material in many regions worldwide (Chen et al., 2022). We previously reported the protection effect of 2'‐FL treatment for food allergies and D‐galactose‐induced aging hallmarks, indicating the immunoregulatory effect and potential application of 2'‐FL for aging‐related metabolic dysfunction in older adults (Kou et al., 2023; Wang et al., 2022). However, the interventive role of 2'‐FL on aging‐induced metabolic disorders and the potential mechanisms that rely on gut microbiome‐T cell immunity remain unclear.

In this study, we validated the hypothesis that 2'‐FL could alleviate aging‐related metabolic disorders by modulating the gut microbial profile‐adaptive immunity axis. The alleviation of dysregulated metabolic indices, including lipid deposition, dyslipidemia, glucose intolerance, systemic inflammation, and gut barrier damage, was confirmed in natural aging mice after 2'‐FL treatment. Flow cytometry analysis revealed the effect of 2'‐FL on T cell differentiation into Tc cells and Th cells and their various subtypes, including Th1, Th2, Th17, and Treg cells. The modulation effect of 2'‐FL treatment on gut microbiota was observed to fundamentally form the connection with T cell immunity and contribute to the identification of the mechanism relying on the microbiome‐adaptive immunity axis. Our findings offer theoretical evidence to support the further application of 2'‐FL to aging‐related metabolic disturbances.

## 2 MATERIALS AND METHODS

### 2.1 Animal treatment

2'‐FL (chemically synthesized, Mw: 488.44 Da, purity >90.8%) was obtained from Glycarbo Co. Ltd., Takamatsu, Japan.

Female C57BL/6J mice (8 and 56‐week‐old) of specific‐pathogen‐free (SPF) grade obtained from Vital River Laboratory (Beijing, China) were subjected to 12 h light/dark cycles, received food and water ad libitum, and then acclimatized for 1 week. The mice at 8 weeks of age were assigned to the young group, and the 56‐week‐old mice were randomly divided into the aged group and the 2'‐FL group (*n* = 6 for each group). The mice in the 2'‐FL group were dosed by oral gavage once daily with 500 mg/kg 2'‐FL for 3 months, and mice in the young group and aged group were administered an equivalent volume of sterile saline. The mice were weighed every 2 weeks, and their survival was monitored daily throughout the experiments. All the animal experiments were approved by the Nankai University Animal Care and Use Committee and were handled according to the Nankai University Animal Welfare Guidelines (protocol code SYXK‐2019‐0001). The 2'‐FL dosage used in this study was established by referring to the daily consumption of infants or children with 2'‐FL from breast milk and the dosage reported in previous studies (Kou et al., 2023).

### 2.2 Oral glucose tolerance test (OGTT)

After the 3‐month 2'‐FL intervention, overnight‐fasted mice were administered a glucose solution (2 g/kg of body mass) by oral gavage, and blood glucose levels were measured at 0, 15, 30, 60, 90, and 120 min after glucose administration. The area under the curve (AUC) was calculated to evaluate glucose tolerance.

### 2.3 Gut permeability assay

An in vivo intestinal permeability assay was performed using fluorescein iso‐thiocyanate (FITC)‐conjugated dextran (Sigma‐Aldrich, St. Louis, USA). Mice were fasted for 6 h and then administered 500 mg/kg of FITC‐dextran via oral gavage. Isoflurane‐anesthetized animals were imaged, and the intestinal fluorescence intensity was measured using an IVIS system (Perkin Elmer, Waltham, USA) 90 min after gastric lavage.

### 2.4 Sample collection

At the end of the experiment, stool samples were collected in sterile tubes and stored at −80 °C until analysis. Blood was collected from the mouse orbit by retro‐orbital venous plexus puncture, centrifuged at 3000 rpm for 20 min, and the serum was collected for biochemical assays. Mice were euthanized by cervical dislocation. Spleen, mesenteric lymph node (MLN), abdominal fat, perirenal fat, liver, and colon samples from the mice were collected, and the weight of the abdominal‐ and perirenal fat was measured.

### 2.5 Serum biochemical measurement

Serum concentrations of total cholesterol (TC), triglycerides (TG), low‐density lipoprotein cholesterol (LDL‐C), high‐density lipoprotein cholesterol (HDL‐C), interleukin‐17A (IL‐17A), interleukin‐17F (IL‐17F), interleukin‐22 (IL‐22), alanine aminotransferase (ALT), and aspartate aminotransferase (AST), and lipopolysaccharide (LPS) were detected using the corresponding kits.

### 2.6 Histological analysis

Liver, abdominal adipose, and colon tissues were collected and fixed overnight at 4°C in a formalin solution. Randomly selected paraffin‐embedded tissue slices from the young, aged, and 2'‐FL groups were stained with hematoxylin and eosin (H&E) and viewed under a light microscope (Nikon Eclipse, Tokyo, Japan). The liver histology was scored based on steatosis, inflammation, and ballooning grades. The degree of colonic injury was assessed using a scale that graded the severity of inflammation, depth of injury, and crypt damage.

### 2.7 Real‐time quantitative PCR (RT‐qPCR)

The mRNA of inflammation‐ and lipid metabolism‐related genes extracted from the liver and colon was used as a template for reverse transcription with subsequent qPCR. The specific primer sequences are listed in Table S1. Relative mRNA expression was calculated by the 2^−ΔΔCt^ method and normalized to β‐actin mRNA levels. The mRNA expression in the samples from the young group served as the control, and mRNA expression in the experimental samples was normalized as a fold change compared to that in young controls.

### 2.8 Flow cytometry analysis

Single‐cell suspensions of the spleen and MLNs were prepared after lysing red blood cells and were resuspended in complete RPMI medium 1640 for cell counting. For analysis of the T cell profile, the isolated cells were then stimulated with a leukocyte activation cocktail plus GolgiPlug (BD Bioscience, NJ, USA) at 37 °C for 6 h. Cells were first stained for surface markers using anti‐CD3e‐FITC, anti‐CD4‐APC, anti‐CD8‐PerCP‐CY5.5, and anti‐CD25‐PE‐CY7 (BD Biosciences, CA, USA). After treatment with the Transcription Factor Fixation/Permeablization Buffer Set (BD Biosciences, CA, USA), cells were stained with anti‐IFN‐γ‐BV650, anti‐IL‐4‐PE, anti‐IL‐17A‐BV786, and anti‐Foxp3‐BV421. Following incubation, the cells were washed twice, resuspended in 500 μL of ice‐cold PBS, and subsequently analyzed using a BD FACS LSRII instrument (BD Biosciences, CA, USA). Data analysis was performed using the FlowJo software (FlowJo, LLC, USA).

### 2.9 Intestinal flora analysis

Fecal microbial DNA was extracted from mouse fecal samples (*n* = 4 mice per group) and subjected to 16S rRNA sequencing. Bacterial sequences were amplified using universal primers for the V3/V4 regions of the 16S rRNA gene (Forward: 5′‐CTACGGGNGGCWGCAG‐3′, Reverse: 5′‐GACTACHVGGGTATCTAATCC‐3′). The purified PCR amplicons were quantified and used as templates for sequencing on the Illumina MiSeq platform (Illumina) according to the protocols of Beijing Novogene. The analysis was performed using the QIIME 2 software pipeline. Alpha diversity indices, including observed species and the Chao1 index, were calculated. Beta diversity, based on the Unweighted UniFrac distance, was visualized using principal coordinates analysis (PCoA) plots. Additionally, a linear discriminant analysis effect size (LEfSe) analysis was conducted to detect gut microbes with significantly differential abundances using the Novogene Cloud platform (LDA score >3.0). The predicted bacterial genes were categorized into functional pathways using the Kyoto Encyclopedia of Genes and Genomes (KEGG) database. Spearman's correlation analysis was used to define the correlations between gut microbiota and metabolic parameters based on the data obtained from all experimental and control groups.

### 2.10 Statistical analysis

The experimental data were expressed as the mean ± SD. All statistical analyses were performed using the GraphPad Prism software (version 9.0; GraphPad Software). A one‐way ANOVA followed by the Newman–Keuls test was used as indicated. Statistical significance was set at *p* < 0.05.

## 3 RESULTS

### 3.1 2'‐FL alleviated aging‐related weight gain and systemic dyslipidemia

**FIGURE 1:**
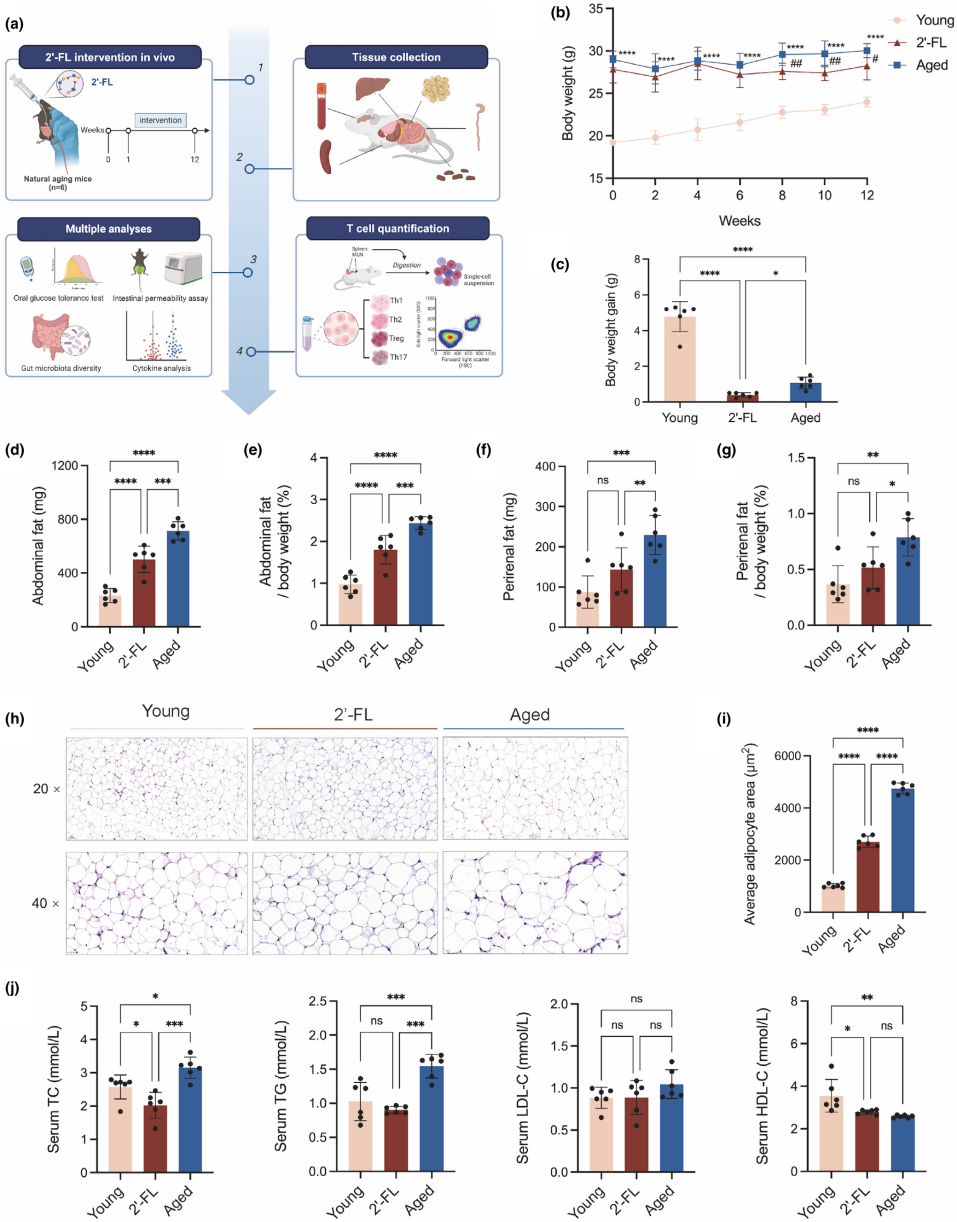
2'‐FL alleviated aging‐related weight gain and systemic dyslipidemia. (a) Schematic representation of the animal model and administration schedule. (b) Body weight changes of mice during the 2'‐FL treatment. Aged versus Young, *p < 0.05, **p < 0.01, ***p < 0.001, ****p < 0.0001; 2'‐FL versus Aged, #*p* < 0.05, ##*p* < 0.01, ###*p* < 0.001, ####*p* < 0.0001. (c) Body weight gain. (d) Abdominal fat weight (e) Abdominal fat weight/ body weight (f) Perirenal fat weight. (g) Perirenal fat weight/body weight. (h) Representative H&E‐stained sections of adipose tissue (i) Quantification of the average adipocyte size. (j) Serum lipid profile. Data are shown as the mean ± SD, and one‐way ANOVA followed by Newman–Keuls test were used (*n* = 6). *p < 0.05, **p < 0.01, ***p < 0.001, ****p < 0.0001.

The metabolic benefits of 2'‐FL were analyzed in mice aged 8 or 56 weeks, as depicted in Figure 1a. The young mice were significantly leaner than the aged mice throughout the experimental period, indicating aging‐associated weight gain. Whereas the body weight of 2'‐FL‐treated mice was retarded considerably from the 8th week of the intervention to the end of the observation period, and a lower level of body weight gain in the 2'‐FL group at the 12th week was also observed compared with that of the aged mice (Figure 1b,c, and Figure S1a). Although skeletal muscle loss was observed in the aged mice, there was no significant difference between the aged and 2'‐FL groups in skeletal muscle mass or its ratio relative to body weight (Figure S1b,c). Higher levels of perirenal fat and abdominal fat and their ratios to the total body weight were noticed in the aged mice when compared to those in the young group, while these obesity traits in the old mice were remarkably reduced with 2'‐FL treatment (Figure 1d–g). As shown in Figure 1h,i, 2'‐FL intervention reversed the aging‐induced increase in adipocyte size (aged vs. young, *p* < 0.0001; 2'‐FL vs. aged, *p* < 0.0001). The abnormal serum lipid profile, characterized by elevated concentrations of TC and TG with a fold of 1.22 and 1.51, respectively, was noticed in aged mice compared with those in their young counterparts, and these metabolic parameters were notably downregulated by the 2'‐FL intervention (Figure 1j). In particular, the TG levels in the 2'‐FL group were down‐regulated to the young state (2'‐FL vs. young, *p* > 0.05). These results demonstrated that 2'‐FL treatment effectively alleviated aging‐related weight gain, fat accumulation, lipid droplet expansion, and systemic dyslipidemia.

### 3.2 2'‐FL relieved aging‐related impaired glucose tolerance and hepatic metabolism

**FIGURE 2:**
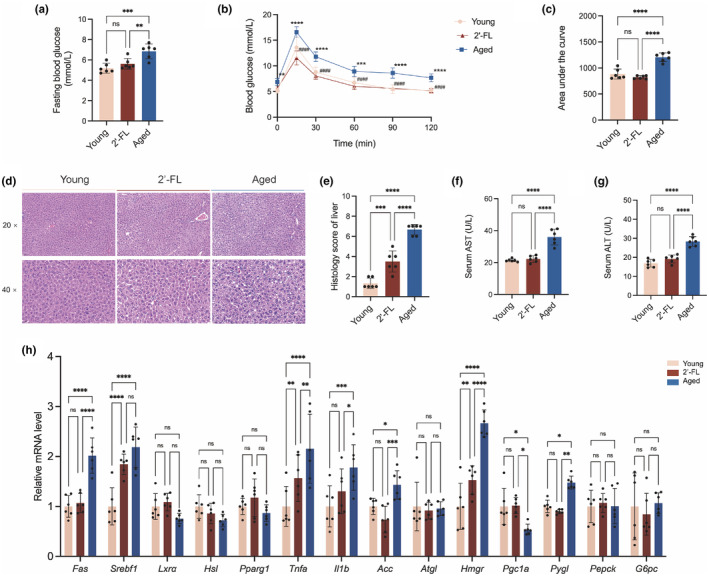
2'‐FL relieved aging‐related impaired glucose tolerance and hepatic metabolism. (a) Fasting blood glucose levels. (b) Oral glucose tolerance test (OGTT). Aged versus Young, *p < 0.05, **p < 0.01, ***p < 0.001, ****p < 0.0001; 2'‐FL versus Aged, #*p* < 0.05, ##*p* < 0.01, ###*p* < 0.001, ####*p* < 0.0001. (c) Area under the curve (AUC). (d) Representative H&E‐stained sections of liver tissues. (e) Histology scores of livers. (f) Serum levels of aspartate aminotransferase (AST). (g) Serum levels of alanine aminotransferase (ALT). (h) Hepatic mRNA expression of glucose and lipid metabolism‐associated genes. Data are shown as the mean ± SD, and one‐way ANOVA followed by Newman–Keuls test were used (*n* = 6). *p < 0.05, **p < 0.01, ***p < 0.001, ****p < 0.0001.

Given that impaired glucose homeostasis during aging is a common abnormal metabolic phenotype, fasting blood glucose levels and glucose tolerance were evaluated in this study. As shown in Figure 2a, aged mice presented significantly elevated fasting blood glucose levels (6.85 mmol/L) when compared with those in young mice (5.18 mmol/L), whereas 2'‐FL treatment resulted in a reduction in plasma glucose concentrations (5.62 mmol/L) with statistical significance. Meanwhile, aged mice supplemented with 2'‐FL showed a greater tolerance to glucose challenge with lower levels of AUC of blood glucose levels (Figure 2b,c). The liver is a key metabolic organ for maintaining normal systemic metabolism as the main site where fasting blood glucose is produced; thus, the regulatory effect of 2'‐FL on liver histology and the mRNA expression of key genes associated with hepatic glucose and lipid metabolism was further assessed. Representative H&E staining of liver tissues showed that the cell membranes of hepatic cells were intact, with well‐defined cell borders, evenly distributed cytoplasm, and a visibly clear nucleus in the young mice (Figure 2d). Liver samples from aged mice were characterized by mild inflammatory infiltration, steatosis, and ballooning degeneration with higher histological scores, compared with the protection of 2'‐FL against steatosis and liver inflammation (Figure 2e). As shown in Figure 2f,g, serum ALT and AST activities recognized as major indicators of hepatotoxicity were significantly inhibited by 2'‐FL intervention in aged mice, and no significant difference was observed when comparing the respective AST and ALT levels between the young and 2'‐FL groups. Moreover, the hepatic mRNA levels of *Fas*, Sterol regulatory element‐binding protein 1C (*Srebp1c*), *Tnfa*, *Il1b*, 1‐aminocyclopropane‐1‐carboxylic acid (*Acc*), HMG‐CoA reductase (*Hmgr*), and liver glycogen phosphorylase (*Pygl*) were markedly upregulated, whereas PPAR‐gamma coactivator 1a (*Pgc1a*) showed significant downregulation in aged mice (Figure 2h). Consistently, 2'‐FL appeared to relieve most of these transcriptional changes in genes associated with metabolic perturbations, presumably by exerting modulatory effects on hepatic glucose and lipid metabolism.

### 3.3 2'‐FL modulated splenic T cell differentiation in aged mice

**FIGURE 3:**
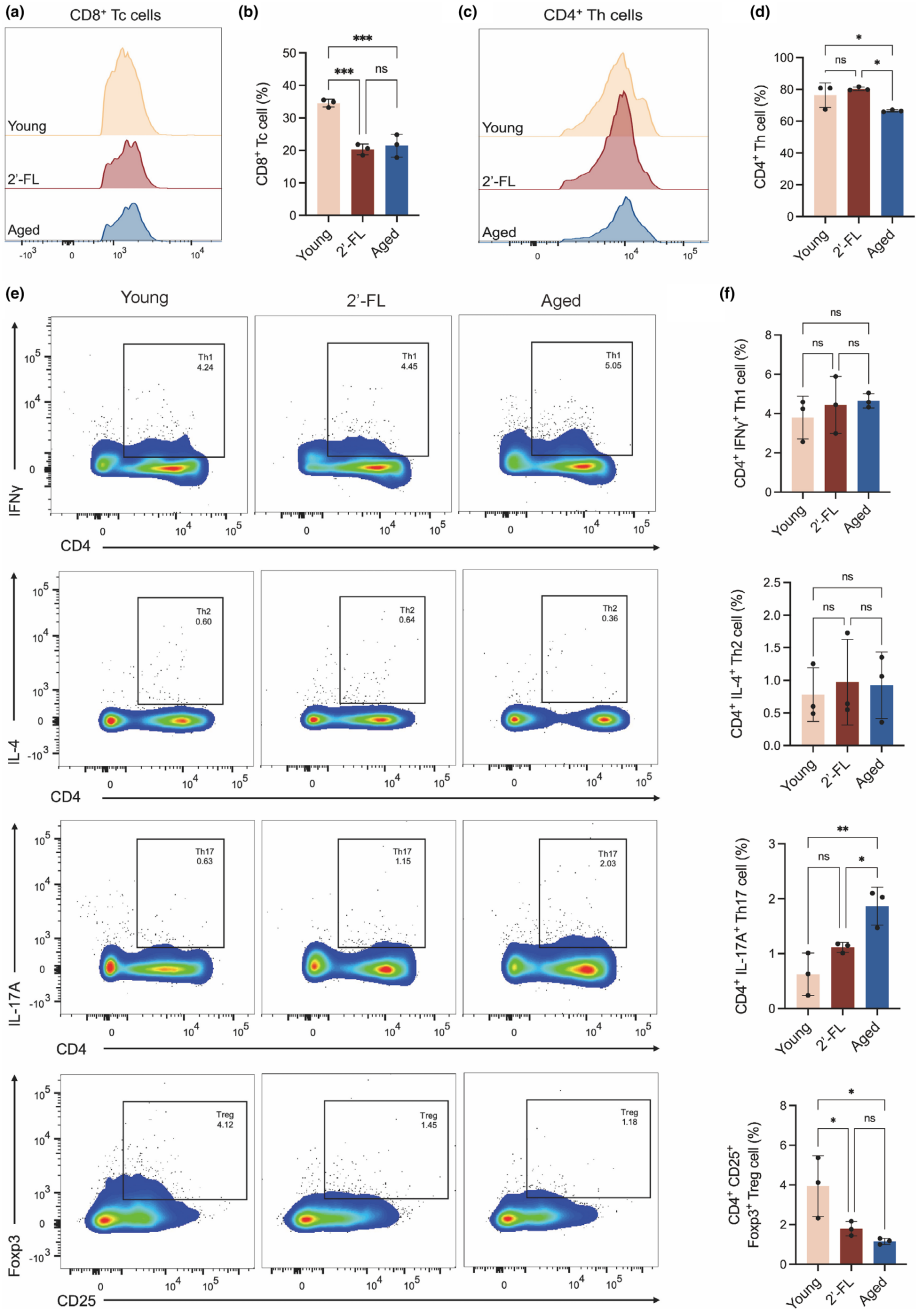
2'‐FL modulated splenic T cell differentiation in aged mice. (a) Representative FACS histograms of CD8^+^ Tc cells. (b) Quantification of splenic CD8^+^ Tc cells (c) Representative FACS histograms of CD4^+^ Th cells. (d) Quantification of splenic CD4^+^ Th cells (e) Representative flow cytometric plots depicting splenic CD4^+^ IFN‐γ^+^ Th1, CD4^+^ IL‐4^+^ Th2, CD4^+^ IL‐17^+^ Th17, and CD4^+^ CD25^+^ Foxp3^+^ Treg cells. (f) The ratio of Th1/Th2, Treg/Th17 cells in spleens. Data are shown as the mean ± SD, and one‐way ANOVA followed by Newman–Keuls test were used (*n* = 3) for flow cytometric analyses. *p < 0.05, **p < 0.01, ***p < 0.001, ****p < 0.0001.

Considering that T cells as major regulators of adaptive immunity have been noted to play critical roles in metabolic homeostasis during aging, the effect of 2'‐FL treatment on the proportion of CD3^+^ CD4^+^ T cells and CD3^+^ CD8^+^ T cells was calculated using FACS histograms and representative flow cytometric plots (Figure 3a–d, Figure S2). The gating strategy for the T cell profile is presented in Figure S3, and the representative flow cytometric plots of the FMO control of Foxp3 staining and the stained samples with no co‐stimulation block treatment are shown in Figure S4, which supports the appropriate positions for cell gating in the flow cytometry analyses. When compared with those in young mice, both CD4^+^ and CD8^+^ splenic T lymphocytes decreased in aged mice (*p* < 0.05), indicating an aging‐induced the imbalance in T cell homeostasis. However, there was a significant improvement in the CD4^+^ Th cell levels with 2'‐FL intervention compared to that in the aged group, but no statistically significant effect of 2'‐FL on CD8^+^ Tc cells was observed. This emphasized that 2'‐FL possibly exerted metabolic benefits through activating CD4^+^ Th cell proliferation rather than CD8^+^ Tc subtypes. Further, the quantification of splenic CD4^+^ Th cell subsets including Th1, Th2, Th17, and Treg cells gated with CD4^+^ IFN‐γ^+^, CD4^+^ IL‐4^+^, CD4^+^ IL‐17^+^, and CD4^+^ CD25^+^ Foxp3^+^, respectively, in response to 2'‐FL treatment in aged mice was then detected (Figure 3e,f). Quantitative analysis revealed an increase in the Th17 cell pool and a decrease in Treg cell levels, accompanied by nonsignificant changes in the frequencies of Th1 and Th2 cells in the aged group, suggesting a pro‐inflammatory T cell phenotype in the senescent microenvironment. Meanwhile, oral administration of 2'‐FL downregulated Th17 cell frequency in aged mice to the level of their young counterparts, but not Treg cell levels, emphasizing that regulation of 2'‐FL on the Th17 pool might be responsible for the alleviation of the impaired metabolism in 2'‐FL‐supplemented aged mice.

### 3.4 2'‐FL improved aging‐induced intestinal Th17/Treg imbalance and barrier compromise

**FIGURE 4:**
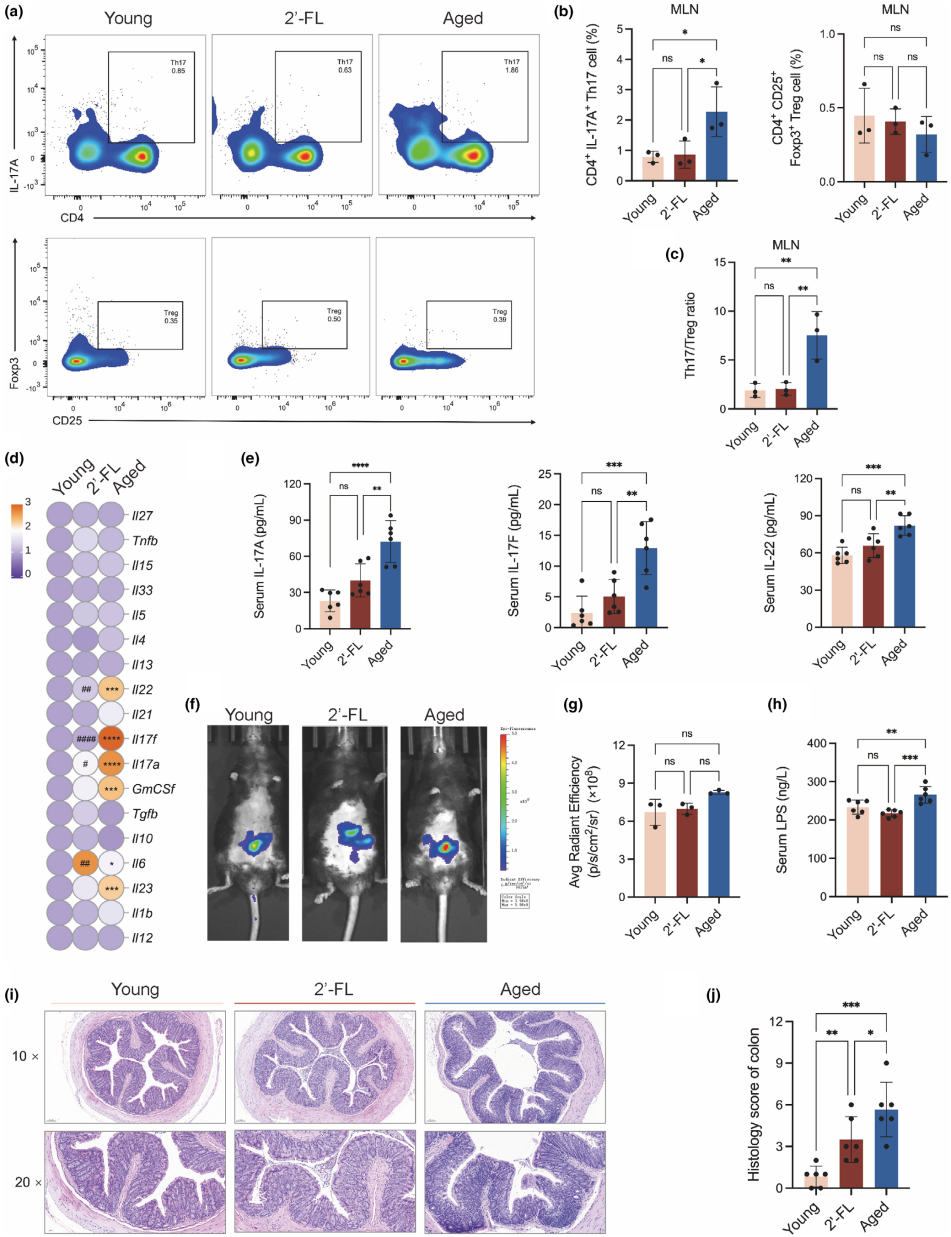
2'‐FL improved aging‐induced intestinal Th17/Treg imbalance and barrier compromise. (a) Representative flow cytometric plots depicting CD4^+^ IL‐17^+^ Th17, and CD4^+^ CD25^+^ Foxp3^+^ Treg cells in MLNs. (b) Quantification of Th17 and Treg cells in MLNs. (c) Th17/Treg ratios in MLNs. (d) Relative mRNA cytokine levels in the colon. (e) Serum levels of IL‐17A. IL‐17F, and IL‐22. (f) Representative images of gut permeability assay under the fluorescent microscope. (g) Fluorescence intensity of FITC‐Dextran in Living imaging. (h) Serum LPS levels. (i) Representative photomicrographs of H&E‐ stained colon tissues. (j) Colonic histological scores. Flow cytometry analysis and in vivo live imaging were conducted in duplicate with three mice per group, and *n* = 6 for other assays. Data are shown as the mean ± SD, and one‐way ANOVA followed by Newman–Keuls test were used. *p < 0.05, **p < 0.01, ***p < 0.001, ****p < 0.0001.

Since 2'‐FL is mainly utilized in the large intestine and is involved in gut adaptive immune responses, the frequency of aging‐induced differential subsets of T cell profiles was quantified in MLNs. As shown in Figure 4a–c, 2'‐FL treatment suppressed both Th17 cell development and higher levels of Th17/Treg ratio in the aged group to the levels of young mice, indicating 2'‐FL might contribute to gut immune homeostasis through inhibiting Th17 differentiation. The mRNA expression of colonic cytokines was measured to further identify the functions of the CD4^+^ T cell subtypes (Figure 4d and Figure S5). The mRNA levels of *Il22*, *Il17f*, *Il17a*, *GmCSf*, *Il6*, and *Il23*, were dramatically enriched in the aged mice compared to those in the young mice, while the aging‐related gene upregulation of *Il17a*, *Il17f*, *and Il22* in the colon was relatively recovered by 2'‐FL supplementation (*p* < 0.05, *p* < 0.0001, *p* < 0.01, *p* < 0.01 for *Il17a*, *Il17f*, and *Il22*, respectively). Correspondingly, the serum circulating concentrations of pro‐inflammatory cytokines released by Th17 cells were then detected, and the results indicated that 2'‐FL treatment dramatically repressed the excessive release of IL17A, IL17F, and IL22 in the aged group and downregulated the levels to the younger state (Figure 4e). The disruption of intestinal integrity is a well‐characterized symptom of aging, and T‐cell imbalance has been reported to accelerate this aging‐induced gut barrier breakage and contribute to the development of metabolic syndrome (Ovadya et al., 2018). In vivo assessment of gut permeability revealed that 2'‐FL slightly reduced the higher levels of FITC‐dextran in the gut of the aged mice, although this effect did not achieve statistical significance (Figure 4f,g). Additionally, there was a significantly higher circulating LPS level in aged mice, whereas 2'‐FL intervention led to a lower LPS release to the levels observed in young mice, indicating the mitigation effect of 2'‐FL on gut barrier damage during aging (Figure 4h). The histological analysis of colon tissues presented a widely disrupted crypt architecture and abundant inflammatory infiltration in the old mice, while 2'‐FL intervention notably improved these colon pathologic damages and pathohistological scoring (Figure 4i,j). These results illustrated that 2'‐FL could exert metabolic benefits through modulation of intestinal immune homeostasis, which emphasized the potential modulation of 2'‐FL on the gut microbiota, and fundamentally contributed to the variation of Th cell differentiation.

### 3.5 2'‐FL remodeled the gut microbial profile in aging mice

**FIGURE 5:**
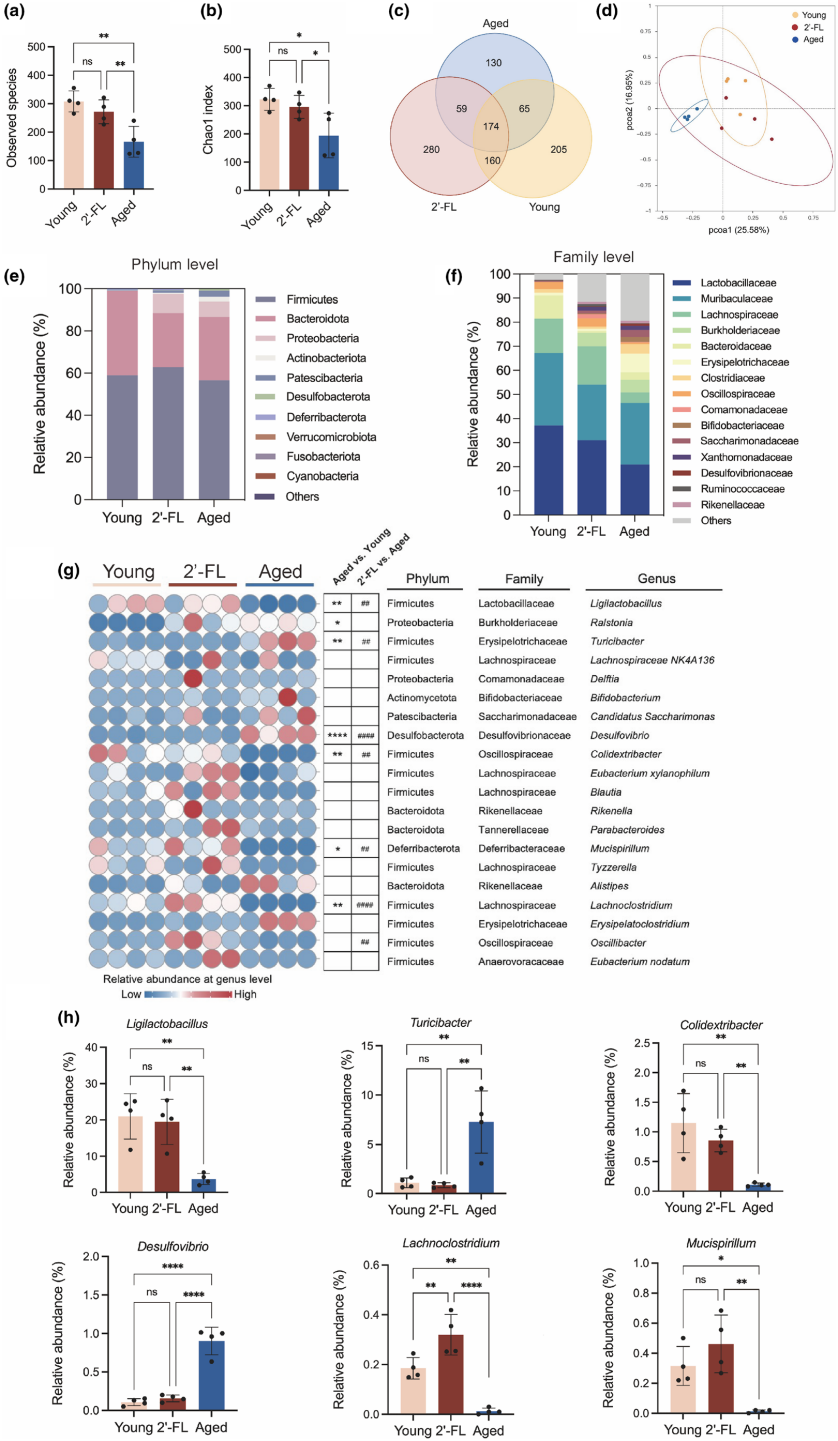
2'‐FL remodeled the gut microbial profile in aging mice. (a) Observed species. (b) Chao1 index. (c) Venn diagram of species concordance among young, aged, and 2'‐FL groups. (d) PCoA (e) Differences of bacterial abundance at the phylum level. (f) Differences of bacterial abundance at the family level. (g) The heatmap of gut microbiota with significant differences in the top 20 at the genus level. Aged versus. Young, *p < 0.05, **p < 0.01, ***p < 0.001, ****p < 0.0001; 2'‐FL versus. Aged, # *p* < 0.05, ## *p* < 0.01, ### *p* < 0.001, #### *p* < 0.0001. (h) Relative abundance of the significantly differential flora at the genus level. Data are shown as the mean ± SD, and one‐way ANOVA followed by Newman–Keuls test were used (*n* = 4 mice per group, randomly selected in each group). *p < 0.05, **p < 0.01, ***p < 0.001, ****p < 0.0001.

Increased intestinal barrier damage has been noted to result in a greater bacterial translocation and gut dysbiosis with age, which subsequently results in deteriorating metabolic performance later in life (Carrasco et al., 2022). Given the interactions between the adaptive immune system and intestinal microbiota, we further explored the influence of 2'‐FL on the gut microbiota composition in elderly mice receiving oral 2'‐FL via 16S rRNA gene sequencing. As shown in Figure 5a,b, a significant decrease in gut microflora α diversity characterized by lowered levels of observed species and Chao 1 index was noticed in the elderly mice compared with that of their younger counterparts, while 2'‐FL upregulated the decreased gut microbial richness with aging (*p* < 0.01, *p* < 0.05, respectively). The Venn diagram displayed that the elderly mice possessed the fewest operational taxonomic units (OTUs) in comparison with young and 2'‐FL‐treated mice, suggesting a potential benefit of 2'‐FL oral administration on gut bacterial diversity (Figure 5c). PCoA demonstrated a significant difference between the young and aged groups regarding gut microbiota composition, whereas 2'‐FL appeared to alter the composition of the gut microbiome in old mice and position it closer to that of the young group (Figure 5d). To further elucidate the impact of 2'‐FL on specific differences in the dominant gut microbes among the young, aged, and 2'‐FL groups, intestinal flora compositions were assessed at the phylum, family, and genus levels (Figure 5e–g). When compared with those in young mice, significantly enriched genera, including *Ralstonia* (Proteobacteria), *Turicibacter* (Firmicutes), and *Desulfovibrio* (Desulfobacterota), were identified, and remarkably downregulated abundances of *Ligilactobacillus* (Firmicutes), *Colidextribacter* (Firmicutes), *Mucispirillum* (Deferribacterota), and *Lachnoclostridium* (Firmicutes) were observed in the aged group. However, the 2'‐FL intervention significantly inhibited the differential changes in the abundance of *Ligilactobacillus*, *Turicibacter*, *Desulfovibrio*, *Colidextribacter*, *Mucispirillum*, and *Lachnoclostridium* during aging, and promoted the enrichment of *Oscillibacter* (Firmicutes) (Figure 5h). These results revealed that 2'‐FL intervention remodeled the gut microbial profile in aged mice.

### 3.6 Correlation analysis between gut microbiome and metabolic indexes

**FIGURE 6:**
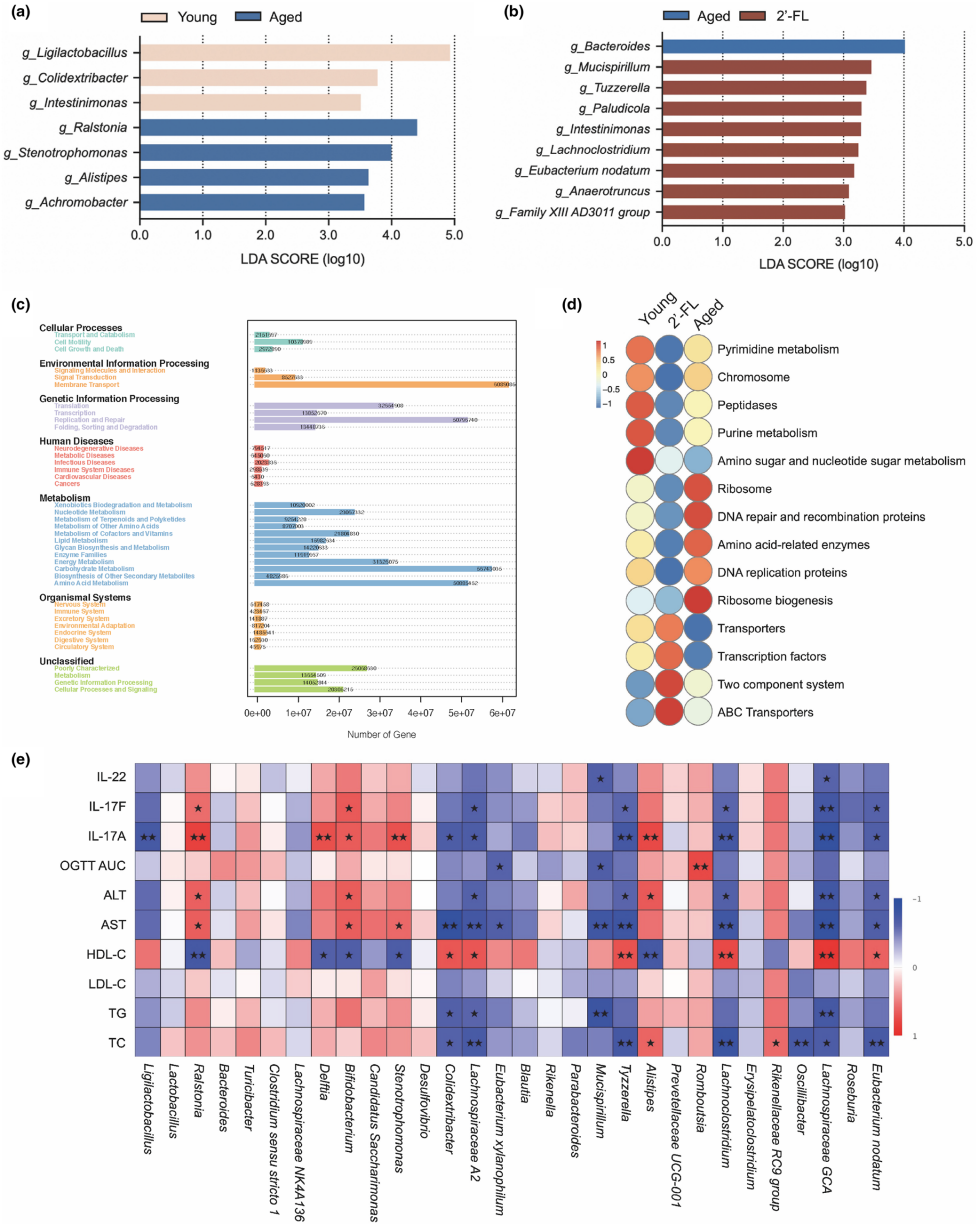
Correlation analysis between gut microbiota and metabolic indexes. (a) LEfSe analyses of fecal genera comparisons between the young and aged groups. (b) LEfSe analyses of fecal genera comparisons between the aged and 2'‐FL groups. (c) KEGG enrichment analysis. (d) Functional prediction of the gut microbiota in different groups. (e) Spearman correlation analysis between gut microbiota and metabolic parameters based on the data obtained from all experimental and control groups. Data are shown as the mean ± SD, and one‐way ANOVA followed by Newman–Keuls test were used (*n* = 4 mice per group, randomly selected in each group). *p < 0.05, **p < 0.01.

As shown in Figure 6a,b, LEfSe analysis was performed to identify the most relevant genera responsible for the differences between the groups (young vs. aged, LDA score >3.5; aged vs. 2'‐FL, LDA score >3.0). Compared with those in young mice, *Ralstonia*, *Stenotrophomonas*, *Alistipes*, and *Achromobacter* were enriched in the aged group, suggesting that these microbes might be linked to poor metabolic outcomes. Meanwhile, the 2'‐FL group showed a clear enrichment in *Mucispirillum*, *Tuzzerella*, *Paludicola*, *Intestinimonas*, *Lachnoclostridium*, *Eubacterium nodatum*, *Anaerotruncus* and *Family XIII AD3011* in contrast to aging mice, and these species may be involved in gut microflora‐mediated alteration of aging‐induced lipid metabolic dysfunction with 2'‐FL treatment. Functional characterization was further validated using the KEGG database, which predicted the interactions between the gut microbiome and host functions, including cellular processes, environmental information processing, genetic information processing, human diseases, metabolism, and organismal systems (Figure 6c). Moreover, 2'‐FL relieved aging‐associated functional changes, including amino sugar and nucleotide sugar metabolism, ribosomes, DNA repair and recombination proteins, Amino acid‐related enzymes, DNA replication proteins, ribosome biogenesis, Transporters, and Transcription factors (Figure 6d). The enriched pathways involving two‐component system and ABC transporters were observed in the 2'‐FL group, which were associated with specific binding, transport, and metabolism of 2'‐FL by targeting strains. To identify specific taxa within the gut microbiota that may modulate host aging‐related metabolic disturbances, a Spearman correlation test between taxa abundances and metabolic parameters was conducted, and the corresponding heatmap is shown in Figure 6e. Among these, the abundance of *Ralstonia*, *Delftia*, *Bifidobacterium*, *Stenotrophomonas*, and *Alistipes* was positively correlated with most of the dysregulated metabolic parameters characterized by higher levels of IL‐17F, IL‐17A, ALT, and AST, coupled with lowered levels of HDL‐C, whereas the growth of *Colidextribacter*, *Lachnospiraceae A2*, *Tyzzerella*, *Lachnoclostridium*, *Lachnospiraceae GCA*, and *Eubacterium nodatum* was negatively correlated with those metabolic dyshomeostasis indices. The correlation analysis confirmed the connection of varied microbes with Th17 cells and identified the potential mechanism of metabolic benefits of 2'‐FL intervention through the microbiome‐adaptive immunity axis.

## 4 DISCUSSION

Increasing evidence suggests that older people are at a higher risk of multiple aging‐related diseases in the context of obesity, and overweight patients have been reported to exhibit an approximately 1.45‐ to 2.76‐fold increased risk of premature deaths compared to those with less adipose (Ng et al., 2017). The gut microbiota has recently been considered a key regulator of organismal senescence and metabolic dysfunction with age and is also known to be primarily influenced by dietary intervention (Zhang, Cheng, & Hu, 2022). Thus, the use of microbiota‐targeted prebiotics in elderly populations with a high burden of metabolic disorders represents a novel strategy to reduce the incidence of aging‐related diseases and improve the healthy life expectancy. The use of 2'‐FL as a new type of prebiotic from human milk oligosaccharide has unique advantages in immunomodulation since it competes to bind pathogens as a decoy receptor and can be preferentially used by beneficial bacteria, including *Bifidobacterium* and *Lactobacilli* (Zhang, Li, et al., 2022). In this study, the role of 2'‐FL in aging‐induced metabolic dysfunction was explored, and the mechanistic basis was revealed based on a mouse model of natural aging. Excess weight gain is often associated with decreased odds of healthy aging and is accompanied by fat deposition and an aberrant lipid profile during aging (Conte et al., 2019; Zheng et al., 2017). Consistently, our findings verified the ameliorative effect of 2'‐FL on aging‐induced metabolic dysfunction characterized by weight gain, adipose deposition, adipocyte expansion, and systemic dyslipidemia. Aging is a primary contributing factor to glucometabolic disorders, with 27% of American adults over 65 years of age diagnosed with diabetes (Nunan et al., 2022). A recent study showed that senescent cell clearance restores glucose tolerance to normal homeostatic levels, indicating a strong association between glucose metabolism and systemic aging (Aguayo‐Mazzucato et al., 2019). This association was consistent with our current study, where an increase in fasting glucose levels and impaired glucose tolerance were observed in old mice but were significantly relieved by 2'‐FL treatment. Given that aging‐induced hepatic steatosis is closely linked with dysregulated glucose homeostasis, the alleviation of 2'‐FL on the hepatic lipid deposition and impaired function was determined in our study.

As aging‐related metabolic dysfunction correlates with changes in various energy metabolism pathways, we further analyzed the expression of genes involved in lipid metabolism in the liver. Of those, a notable down‐regulation of hepatic *Pgc1a* mRNA was observed in aged mice, following previous research where aging‐induced oxidative damage accumulation was attenuated in PGC1α‐transgenic mice relative to wild‐ type (Tsunemi et al., 2012). This differential mRNA level of *Pgc1a* was repressed by 2'‐FL intervention, suggesting its potential role in aging‐driven lipid metabolism disorders. In addition, our findings showed that 2'‐FL markedly inhibited the aging‐induced mRNA overexpression of *Fas*, *Acc*, and *Hmgr*, which are recognized as regulators of both lipid metabolism and aging. Specifically, a high‐fat‐diet‐induced increase in *Fas* has been shown to promote insulin resistance and is positively correlated with aging hallmarks, whereas *Acc*, the rate‐limiting enzyme in fatty acid synthesis, plays a paramount role in fatty acid synthesis and contributes to systematic aging (Currais et al., 2019; Wueest et al., 2014). *Hmgr*, a rate‐limiting enzyme in the biosynthesis of cholesterol, was found to be elevated in both obese and aged mice, which is in agreement with the results of our study (Machado et al., 2005). In parallel, the mRNA expression of key regulators of glycogen metabolism, including *Pygl*, was elevated in aged mice and inhibited by 2'‐FL treatment, which balanced the glucose supply (Li et al., 2022). Moreover, it is well known that systemic inflammation increases with age and is responsible for disrupted lipid homeostasis (Furman et al., 2019). Consistently, higher mRNA levels of inflammatory factors, including *Tnfa* and *Il1b*, were noticed in aged mice and were significantly downregulated after the 2'‐FL intervention, suggesting an improvement in aging‐related inflammation.

Although aging‐driven inflammation was originally ascribed to the prolonged accumulation of non‐immune senescent cells, mounting reports have recently highlighted the central role of T cells and have started to describe the causal contribution of the T cell profile to both the etiology and progression of aging‐induced diseases (Mogilenko et al., 2021). In our study, a reduction of splenic CD4^+^ Th cells and CD8^+^ Tc cells was identified, which may partially account for the inflammation and glucose intolerance in aged mice because CD8^+^ Tc cells are mainly responsible for fighting pathogen infections and inhibiting proinflammatory phenotypes via the secretion of perforin, and CD4^+^ Th cells have been reported to highly correlate with glucose tolerance and insulin sensitivity (Revelo et al., 2015; Winer et al., 2009). However, this aging‐induced lower proportion of the CD4^+^ T cells was relieved in the 2'‐FL‐treated mice, thus multiple T cell subsets including Th1, Th2, Th17, and Treg cells gated with CD4^+^ IFN‐γ^+^, CD4^+^ IL‐4^+^, CD4^+^ IL‐17^+^, and CD4^+^ CD25^+^ Foxp3^+^, respectively, were further quantified. Our results demonstrated that aged mice exhibited elevated percentages of Th17 cells and reduced percentages of Treg cells, in line with previous studies in which the senescence‐associated secretory phenotype tilted the balance in favor of developing a Th17 phenotype, exacerbated inflammation, and contributed to age‐related metabolic dysregulation, while lower levels of Treg had significant negative consequences for metabolic homeostasis (Bertola et al., 2012; Deng et al., 2017). The 2'‐FL treatment effectively suppressed the Th17 cell enrichment in the old mice, emphasizing the protective role of 2'‐FL in aging‐induced metabolic disorders, possibly through the complex interplay involving Th17 rather than Treg cells in the senescent immune microenvironment.

During aging, increased intestinal permeability is a well‐defined phenomenon accompanied by impaired gut barrier function and gut dysbiosis, which further leads to exacerbated inflammation and metabolic disorders (Santoro et al., 2021). These aging‐induced changes in gut physiology are correlated with a Th17/Treg imbalance and multiple cytokine abnormalities, of which IL‐17 and IL‐22 exert well‐recognized regulatory effects on adiposity as inflammatory mediators of innate and adaptive immune responses (Dalmas et al., 2014). Correspondingly, our findings identified a higher Th17/Treg ratio, excess release of Th17‐released inflammatory factors, including IL‐17A, IL‐17F, and IL‐22, elevated intestinal permeability, and colon pathological injury in the senescent gut, while 2'‐FL intervention alleviated the aging‐related disruption of gut immune homeostasis.

Commensal microorganisms have intricate and co‐occurring relationships with their hosts that sustain a broad range of intestinal physical barriers and immunological responses, whereas aging‐induced dysregulation of gut immune homeostasis can, in turn, amplify gut microbial dysbiosis (Thevaranjan et al., 2017). A deeper insight into the interaction between dietary 2'‐FL intervention and gut microbiota is critical for understanding the regulatory mechanisms of this novel prebiotics on metabolic health in older people. Our study highlighted that the lowered levels of taxonomic diversity of microorganisms were significantly mitigated by 2'‐FL intervention. Since it is urgently needed to define 2'‐FL‐mediated functional gut microbes that benefit host metabolic health, further analysis of the changes in the microbial composition of each group was performed, where 2'‐FL promoted the growth of beneficial genera including *Ligilactobacillus*, *Colidextribacter*, *Mucispirillum*, and *Lachnoclostridium* and inhibited the abundance of *Turicibacter*, *and Desulfovibrio* in aged mice. This is consistent with previous findings in which *Ligilactobacillus* intervention improved the fecal inflammatory state in older adult residents and was significantly associated with the Th17/Treg ratio (Mozota et al., 2021; Qin et al., 2023). *Colidextribacter* has been demonstrated to alleviate the gut inflammation, in line with our findings, and the enrichment of *Colidextribacter* was noticed in the 2'‐FL‐supplemented mice and negatively correlated with disturbed metabolic features (Niu et al., 2021). The correlation of microbes with Th17‐secreted cytokines confirmed the involvement of certain microbes in 2'FL‐mediated immunomodulation and intervention in aging‐related metabolic disorders. Additionally, *Mucispirillum* and *Lachnoclostridium* were enriched in the 2'‐FL group and had a negative correlation with dysregulated metabolic indices, suggesting that these two genera might be potential targets for the regulatory effect of 2'‐FL on aging‐induced metabolic disorders. In agreement with our findings, a correlation between the abundance of *Mucispirillum* and *Lachnoclostridium* and Th17/ Treg balance with dietary intervention has been noted in previous studies (Chen et al., 2021; Zhang, Gu, et al., 2022). As indicated in our microbial analysis, the abundance of the senescence‐related strains *Desulfovibrio* and *Turicibacter* was elevated in aged mice but repressed by 2'‐FL treatment, which is consistent with previous studies showing that these two pathogens can be used to predict immune‐related adverse events and contribute to Th17/Treg imbalance (Hamada et al., 2023; Song et al., 2022).

This study has certain limitations regarding the long‐term effects and sex differences in the protective effects of 2'‐FL on aging‐related metabolic disorders. Whether long‐term 2'‐FL treatment would result in a more profound beneficial effect remains to be examined in subsequent work, and more attention should be paid to sex issues to provide a widely applicable approach that might help improve the metabolic health during aging. Nonetheless, our findings revealed a key role of 2'‐FL in relieving aging‐related metabolic derangement characterized by body weight gain, hyperlipidemia, disrupted hepatic metabolism, fat deposition, and glucose intolerance. The statistical analysis between groups of young and 2'‐FL dominantly revealed the significant differences in their metabolic status, which potentially indicated the ameliorative effect of 2'‐FL on aging‐related metabolic disorders rather than restoring to the young state. The potential mechanisms may involve the improvements in pathological colonic damage, gut microbial dysbiosis, and intestinal immune homeostasis by inhibiting the overactivation of Th17 cells, regulating inflammatory cytokine release, and remodeling the Th17/Treg balance. These findings provide theoretical support for the development of a 2'‐FL intervention to alleviate aging‐induced metabolic disorders, and further offer a novel gut microbiota‐targeted strategy for health decline in older people.

## AUTHOR CONTRIBUTIONS

**Ang Li**: Investigation; formal analysis; writing‐original draft. **Ruixin Kou**: Methodology. **Ruishan Wang**: Resources. **Jin Wang**: Funding acquisition. **Bowei Zhang**: Data curation. **Jingmin Liu**: Software. **Yaozhong Hu**: Writing‐review and editing. **Shuo Wang**: Project administration and supervision. All authors agree to be accountable for the content of the work and approved the manuscript.

## CONFLICT OF INTEREST STATEMENT

The authors declare that the research was conducted in the absence of any commercial or financial relationships that could be construed as a potential conflict of interest.

## Supporting information


Data S1.

## Data Availability

The data that support the findings of this study are available from the corresponding author upon reasonable request.
