# Supplementary material for: 2'‐Fucosyllactose attenuates aging‐related metabolic disorders through modulating gut microbiome‐T cell axis
Source: Aging Cell. 2024 Sep 20;24(1):e14343. doi: 10.1111/acel.14343 (PMC11709090; doi:10.1111/acel.14343)
Supplement: Supplementary file 1 — Data S1. [file ACEL-24-e14343-s001.docx]

**2’-Fucosyllactose attenuates aging-related metabolic disorders through modulating gut microbiome-T cell axis**

*Ang Li^1^, Ruixin Kou^1^, Ruishan Wang^1^, Jin Wang^1^, Bowei Zhang^1^, Jingmin Liu^1^, Yaozhong Hu^1, *^, Shuo Wang^1, *^*

^1^ Tianjin Key Laboratory of Food Science and Health, School of Medicine, Nankai University, Tianjin, China

angli@mail.nankai.edu.cn (A. L.), [kourx@mail.nankai.edu.cn](mailto:kourx@mail.nankai.edu.cn) (R. K.), w18103658595@163.com (R. W.), wangjin@nankai.edu.cn (J. W.), bwzhang@nankai.edu.cn (B. Z.), liujingmin@nankai.edu.cn (J. L.), yzhu@nankai.edu.cn (Y. H.), [wangshuo@nankai.edu.cn](mailto:wangshuo@nankai.edu.cn) (S. W.)

*** Correspondence**

Shuo Wang, Yaozhong Hu, Tianjin Key Laboratory of Food Science and Health, School of Medicine, Nankai University, Tianjin 300071, China.

Emails: wangshuo@nankai.edu.cn (S. W.), yzhu@nankai.edu.cn (Y. H.)

**Table S1.** Primer sequences for qPCR

| Gene | Forward primer（5’→3’） | Reverse primer（5’→3’） |
| --- | --- | --- |
| *Il10* | GCTGGACAACATACTGCTAACC | ATTTCCGATAAGGCTTGGCAA |
| *Il12* | GTCCTCAGAAGCTAACCATCTCC | CCAGAGCCTATGACTCCATGTC |
| *Il13* | CAGCCTCCCCGATACCAAAAT | GCGAAACAGTTGCTTTGTGTAG |
| *Tgfb* | CTTCAATACGTCAGACATTCGGG | GTAACGCCAGGAATTGTTGCTA |
| *Tnfb* | CATTCCCACTCCCATCTACC | GTCCTTGAAGTCCCGGATAC |
| *Il27* | CTGTTGCTGCTACCCTTGCTT | CTCCTGGCAATCGAGATTCAG |
| *Il15* | CATCCATCTCGTGCTACTTGTG | GCCTCTGTTTTAGGGAGACCT |
| *Il33* | ATTTCCCCGGCAAAGTTCAG | AACGGAGTCTCATGCAGTAGA |
| *Il5* | TCAGGGGCTAGACATACTGAAG | CCAAGGAACTCTTGCAGGTAAT |
| *Il4* | GGTCTCAACCCCCAGCTAGT | GCCGATGATCTCTCTCAAGTGAT |
| *Il22* | ATGAGTTTTTCCCTTATGGGGAC | GCTGGAAGTTGGACACCTCAA |
| *Il21* | GGACCCTTGTCTGTCTGGTAG | TGTGGAGCTGATAGAAGTTCAGG |
| *Il17f* | TGCTACTGTTGATGTTGGGAC | CAGAAATGCCCTGGTTTTGGT |
| *Il17a* | TTTAACTCCCTTGGCGCAAAA | CTTTCCCTCCGCATTGACAC |
| *GmCSf* | GGCCTTGGAAGCATGTAGAGG | GGAGAACTCGTTAGAGACGACTT |
| *Il23* | AATAATGTGCCCCGTATCCAGT | GCTCCCCTTTGAAGATGTCAG |
| *Hsl* | GCTAGCCAGGCTCATCTCCT | GTTCTTGAGGTAGGGCTCGT |
| *Fas* | GCTGCGGAAACTTCAGGAAAT | AGAGACGTGTCACTCCTGGACTT |
| *Hmgr* | TGCCTGGATGGGAAGGAGTA | GCACCTCCACCAAGGCTTAT |
| *Atgl* | ACAGCTCCAACATCCAC | AGCCCTGTTTGCACATCTCT |
| *Lxra* | TCAGAAGAACAGATCCGCTTG | CGCCTGTTACACTGTTGCT |
| *Pparg1* | CCAGCATTTCTGCTCCACAC | ATTCTTGGAGCTTCAGGCCA |
| *Acc* | GGCAGCAGTTACACCACATAC | TCATTACCTCAATCTCAGCATAGC |
| *Srebf1* | CTGGTGAGTGGAGGGACCAT | GACCGGTAGCGCTTCTCAAT |
| *Pgc1a* | AGCCGTGACCACTGACAACGAG | GCTGCATGGTTCTGAGTGCTAAG |
| *Pepck* | AGCATTCAACGCCAGGTTC | CGAGTCTGTCAGTTCAATACCAA |
| *G6pc* | AAAAAGCCAACGTATGGATTCCG | CAGCAAGGTAGATCCGGGA |
| *Pygl* | CGACGGCAGATCAGCATCC | GAAGTGCAGGTGACGGTTGAA |
| *Tnfa* | AATGGCCTCCCTCTCATCAG | CCACTTGGTGGTTTGCTACG |
| *Il6* | TAGTCCTTCCTACCCCAATTTCC | TTGGTCCTTAGCCACTCCTTC |
| *Il-1b* | AAGGGCTG TTCCAAACCTTTGAC | TGCCTGAAGCT TTGTTGATGTGC |
| *β-actin* | ACAGCAGTTGGTTGGAGCAA | ACGCGACCATCCTCCTCTTA |

**
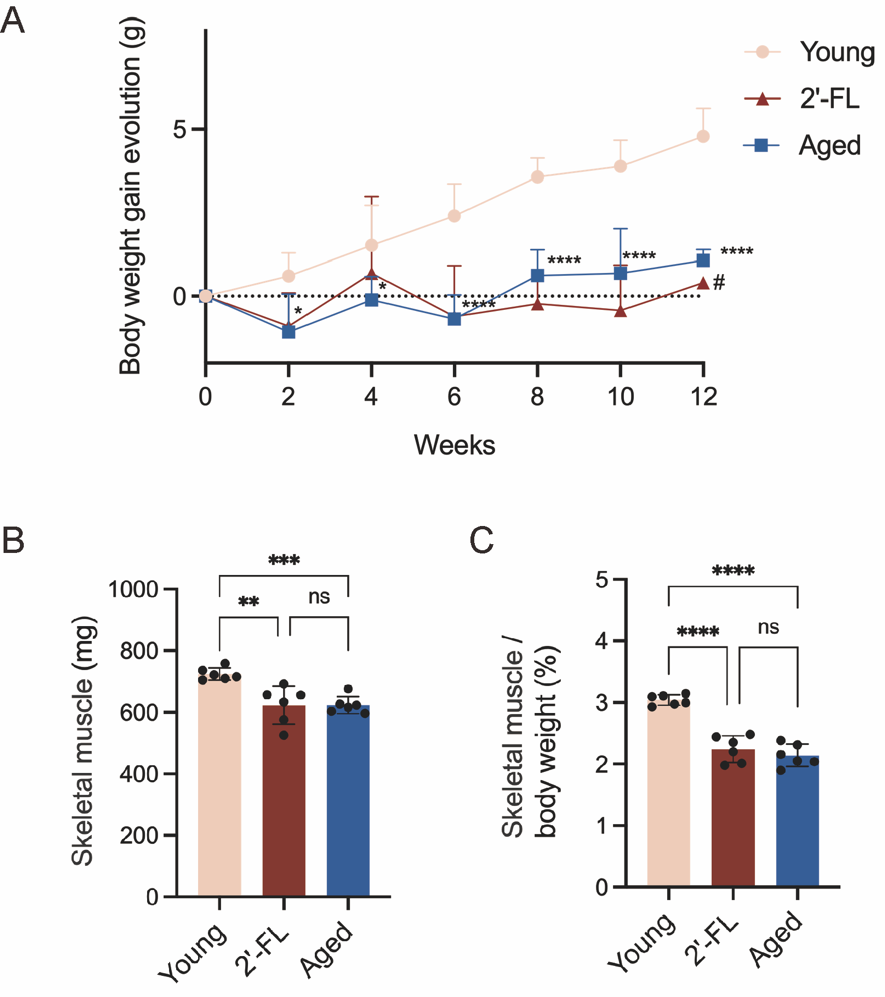
**

**Figure S1.** Body weight gain evolution and aging-related skeletal muscle loss. (A) Body weight gain evolution. Aged vs Young, * p <0.05, ** p <0.01, *** p <0.001, **** p <0.0001; 2’-FL vs Aged, # p <0.05, ## p <0.01, ### p <0.001, #### p <0.0001. (B) Skeletal muscle weight. (C) Skeletal muscle weight/body weight. Data are shown as the mean ± SD, and one-way ANOVA followed by Newman-Keuls test were used (n = 6). * p <0.05, ** p <0.01, *** p <0.001, **** p <0.0001.


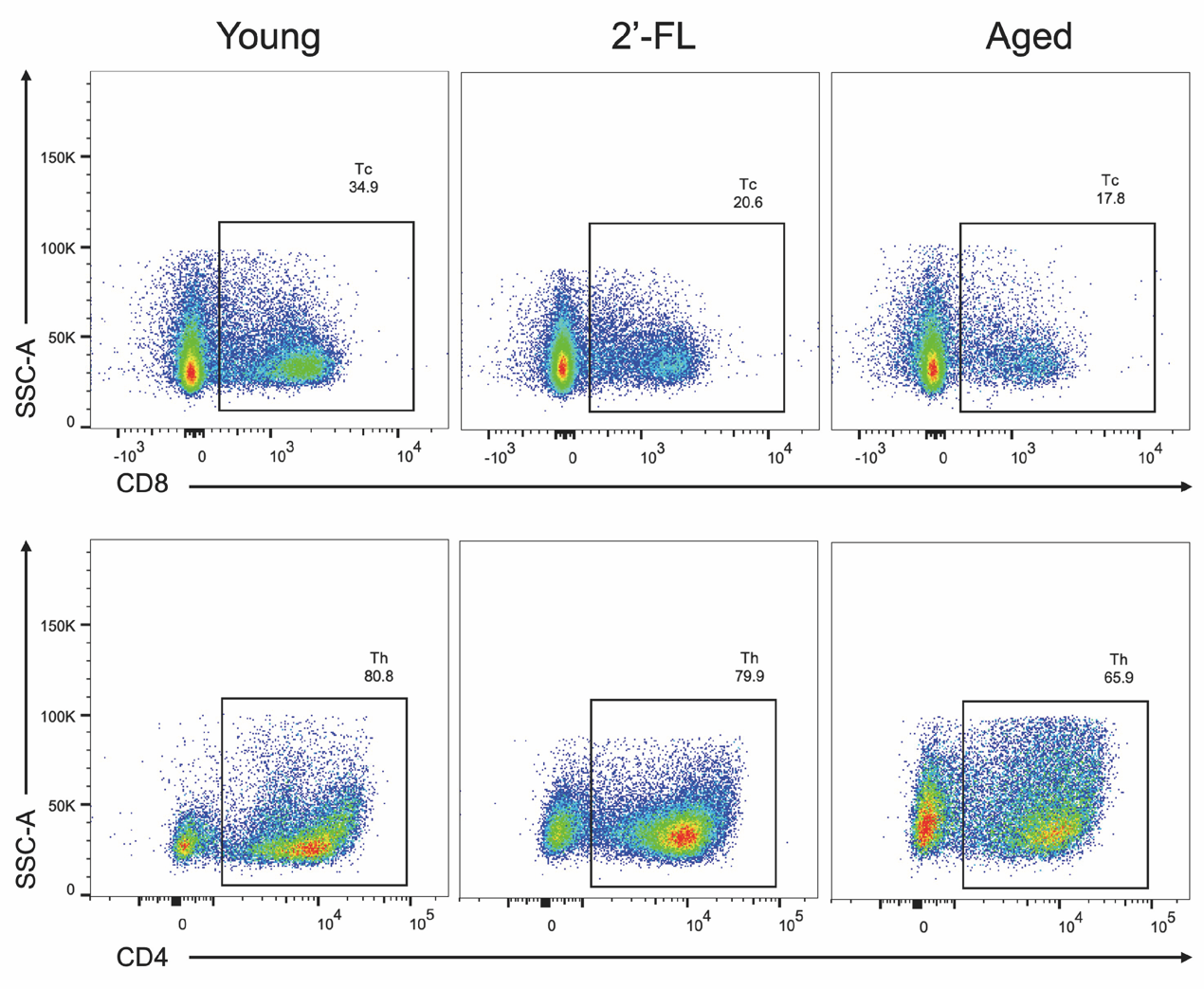


**Figure S2.** Representative flow cytometric plots depicting splenic CD3^+^ CD8^+^ Tc cells, and CD3^+^ CD4^+^ Th cells.


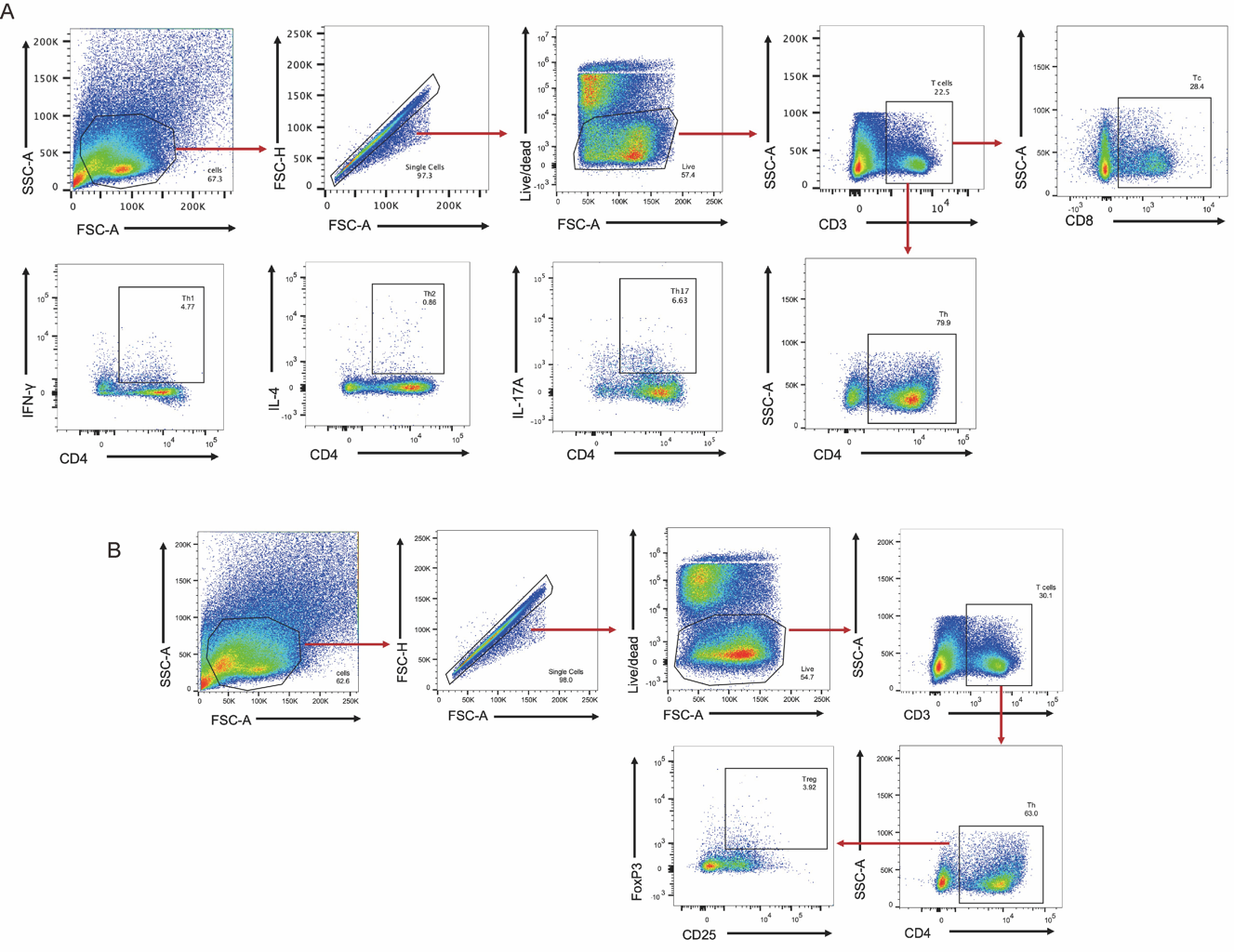


**Figure S3.** Gating strategy of the flow cytometry results of T cell subsets. (A) Gating strategy of CD3^+^ CD4^+^ Th cells, CD3^+^ CD8^+^ Tc cells, CD4^+^ IFN-γ^+^ Th1 cells, CD4^+^ IL-4^+^ Th2 cells, CD4^+^ IL-17^+^ Th17 cells. (B) Gating strategy of CD4^+^ CD25^+^ Foxp3^+^ Treg cells.

**
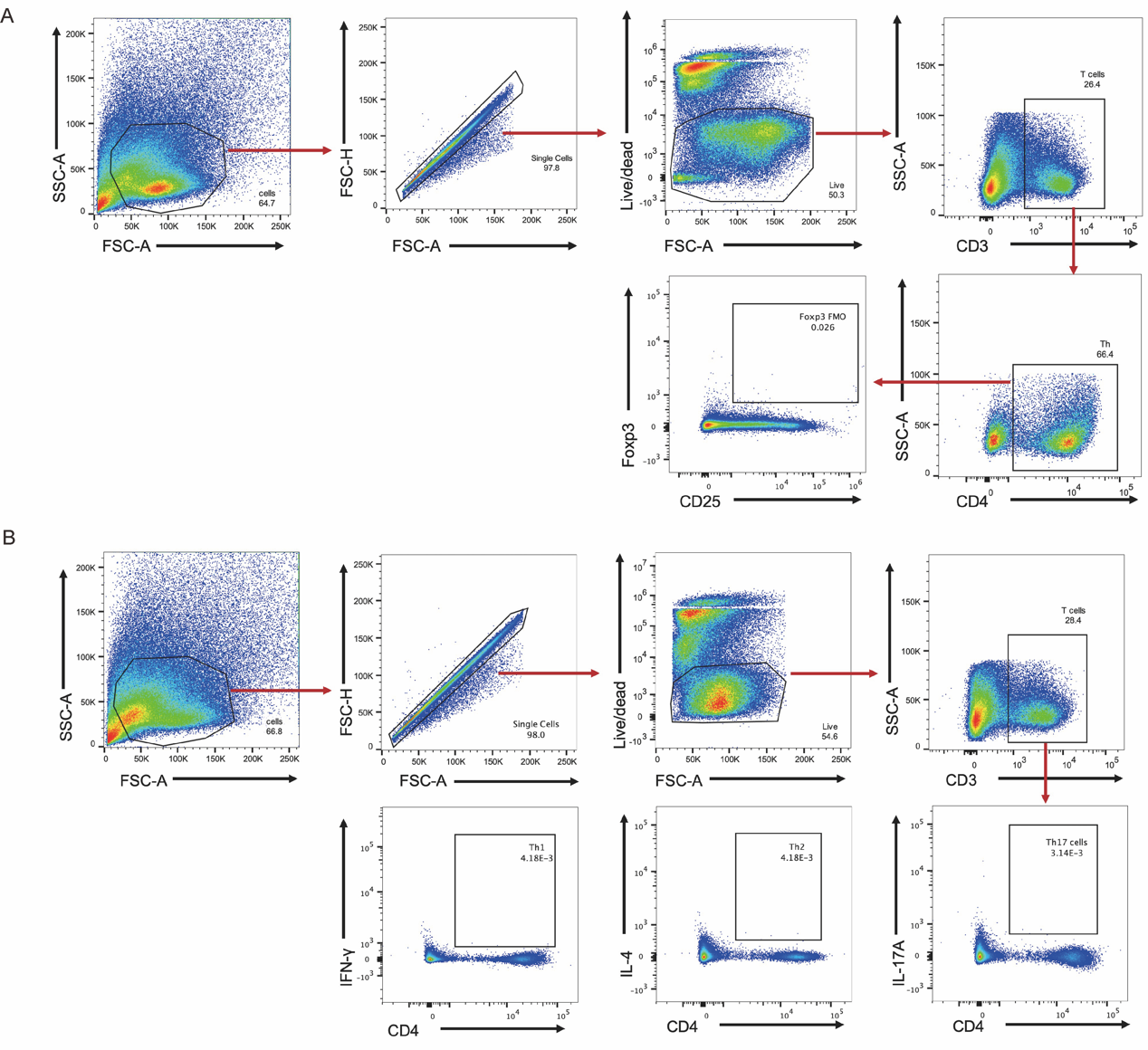
**

**Figure S4.** Gating strategy of the flow cytometry results of control samples. (A) Gating strategy of Foxp3 FMO control. (B) Gating strategy of the stained cells with no co-stimulation block treatment.


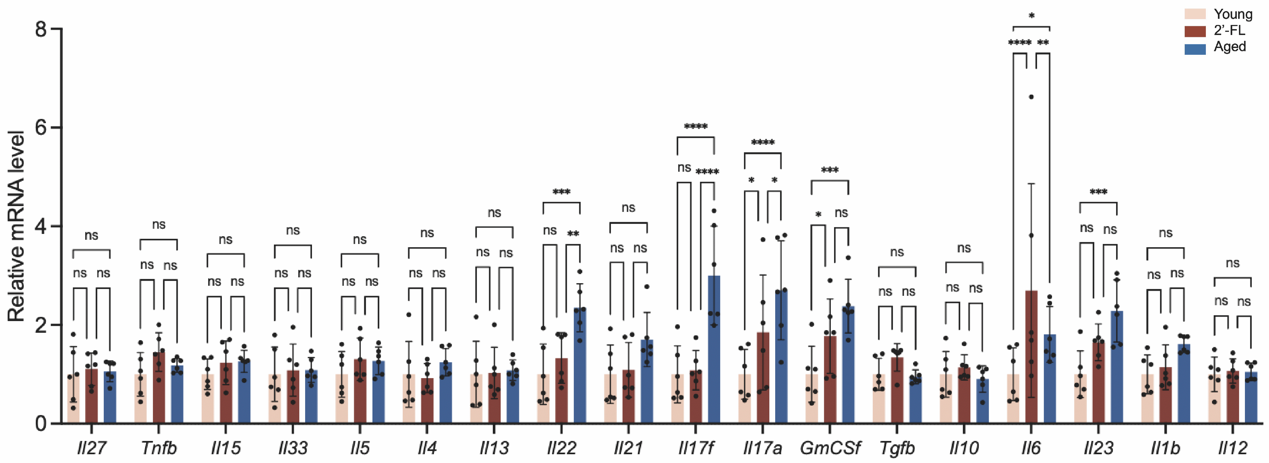


**Figure S5.** The mRNA expression of colonic cytokines. Data are shown as the mean ± SD, and one-way ANOVA followed by Newman-Keuls test were used (n = 6). * p <0.05, ** p <0.01, *** p <0.001, **** p <0.0001.
